# Quorum Sensing-Mediated and Growth Phase-Dependent Regulation of Metabolic Pathways in *Hafnia alvei* H4

**DOI:** 10.3389/fmicb.2021.567942

**Published:** 2021-03-02

**Authors:** Congyang Yan, Xue Li, Gongliang Zhang, Yaolei Zhu, Jingran Bi, Hongshun Hao, Hongman Hou

**Affiliations:** ^1^School of Food Science and Technology, Dalian Polytechnic University, Dalian, China; ^2^Liaoning Key Lab for Aquatic Processing Quality and Safety, Dalian, China

**Keywords:** *Hafnia alvei*, luxI/R-mediated QS, growth phase-dependent manner, transcriptome, metabolism

## Abstract

Quorum sensing (QS) is a widespread regulatory mechanism in bacteria used to coordinate target gene expression with cell density. Thus far, little is known about the regulatory relationship between QS and cell density in terms of metabolic pathways in *Hafnia alvei* H4. In this study, transcriptomics analysis was performed under two conditions to address this question. The comparative transcriptome of *H. alvei* H4 wild-type at high cell density (OD_600_ = 1.7) relative to low cell density (OD_600_ = 0.3) was considered as growth phase-dependent manner (GPDM), and the transcriptome profile of *luxI/R* deletion mutant (Δ*luxIR*) compared to the wild-type was considered as QS-mediated regulation. In all, we identified 206 differentially expressed genes (DEGs) mainly presented in chemotaxis, TCA cycle, two-component system, ABC transporters and pyruvate metabolism, co-regulated by the both density-dependent regulation, and the results were validated by qPCR and swimming phenotypic assays. Aside from the co-regulated DEGs, we also found that 59 DEGs, mediated by density-independent QS, function in pentose phosphate and histidine metabolism and that 2084 cell-density-dependent DEGs involved in glycolysis/gluconeogenesis and phenylalanine metabolism were influenced only by GPDM from significantly enriched analysis of transcriptome data. The findings provided new information about the interplay between two density-dependent metabolic regulation, which could assist with the formulation of control strategies for this opportunistic pathogen, especially at high cell density.

## Introduction

Quorum sensing (QS) is a cell to cell communication system that allows bacteria to coordinate gene expression in response to cell density, which is mediated by diffusible chemical signals such as acyl-homoserine lactones (AHLs) (Goo et al., 2015). Generally, the concentration of AHLs increases as cell density increases, leading to the coordinated expression of various genes when the concentration of AHLs reaches a certain level (Mellbye et al., 2016). The phenotypes associated with QS-controlled genes are often required for survival and/or virulence in several bacteria, such as motility, biofilm formation, colonization, adhesion, virulence factor secretion and nutrient acquisition (Goo et al., 2015; Khider et al., 2019). The QS controlled activities prove costly and ineffective when bacterial densities are low, while it become more beneficial when carried out by a group (Pai et al., 2012). Therefore, the QS system allows bacteria to switch metabolic direction between high cell density (HCD) and low cell density (LCD).

However, cell density is also important for bacterial metabolism. A number of instances have been reported where the regulation of growth phase-dependent manner (GPDM) correlates with some physiological process in microorganisms, including food spoilage (Gram et al., 2002; Joffraud et al., 2006), secretion of virulence factors (Albuquerque et al., 2013; Doekes et al., 2019), biofilm formation (Khider et al., 2019) and chemotaxis (Colin et al., 2019). For example, *Escherichia coli* moderately enhance chemotactic drift at intermediate cell densities, and then strongly suppresses it at HCD (Colin et al., 2019). Conversely, the expression of ribosome biogenesis genes is highest during HCD, while lipogenic proteins are more highly expressed later in the yeast growth cycle (Blank et al., 2017). Additionally, the increase of cell density can significantly increase the expression of *hcnABC* (hydrogen cyanide synthase genes), and reach its optimal levels during the transit from exponential to stationary growth phase of *Pseudomonas aeruginosa* (Castric et al., 1979). Hence, as for the above density-dependent regulation, QS and GPDM, our understanding of how they influence the direction of metabolism is in its infancy.

In our previous study, we have reported that the QS systems of *Hafnia alvei* H4 consists of AHLs synthase encoded by *luxI* gene and AHLs homologous receptors encoded by *luxR* gene (Li X. et al., 2019), which involved in the density-dependent biological processes including food spoilage and virulence formation (Blana et al., 2017; Li T. T. et al., 2019), through transcription regulation of target genes via AHL-LuxR dimerization-mediated signaling cascades. Thus, in this study, we used this bacterium as a model organism, and compared and analyzed the transcriptome data of its *luxI/R*-mediated QS and GPDM to exploit the influence of these regulation on the metabolism and to gain insight into the favorable control of density-dependent metabolic pathways.

## Materials and Methods

### Bacterial Strains and Culture Conditions

The deletion of *luxI/R* gene (Δ*luxIR*) was generated in our previous study (Zhu et al., 2019). *H. alvei* H4 wild-type and Δ*luxIR* were initially grown for 12 h in Luria-Bertani (LB) medium at 150 rpm and 30°C, and subcultured 1:100 into fresh medium overnight. For pigment production assay as described by Zhu et al. (2019), briefly, supernatants were collected at indicated time by centrifugation, and an equal volume of ethyl acetate containing 0.1% acetic acid (v/v) was added to the fluid. The mixture was incubated at 25°C for 2.5 h with shaking at 150 rpm, and then, the organic phases was removed and freeze-dried under vacuum. The residue was dissolved in 1 ml ultra-pure water. 60 μl of the AHLs extract was dispensed into the plates containing 20 ml LB agar medium and 5 ml overnight cultures of biosensor CV026. The plates were incubated at 30°C until a purple zone could be observed around the point of AHL application.

### Preparation of *H. alvei* H4 Transcriptome Samples

Luria-Bertani medium (100 ml) was inoculated with an overnight cultures of wild-type *H. alvei* or Δ*luxIR*, and then cultivated as batch cultures for 12 h at 30°C with shaking at 150 rpm (marked as W12 and IR12, respectively). The cultures were separated into 50-ml fractions at indicated time points, which were subsequently transferred into centrifuge tubes, chilled on ice, and centrifuged at 8,000 × *g* for 10 min. The supernatant of each sample was discarded, and the cell pellets were directly frozen in liquid nitrogen and stored at −80°C until further use. The preparation of the W2 sample followed the same protocol except that the culturing time was 2 h (marked as W2).

### Transcriptome Sequencing

Total RNA was extracted from W2, W12, and IR12 with an RNAprep pure Cell/Bacteria Kit (Tiangen Biotech, Beijing, China) according to the manufacturer’s instructions, and the mRNA was purified using Ribo-Zero rRNA Removal Kit (Bacteria) (Illumina). First-strand cDNA synthesis was performed using random hexamer primer and M-MuLV Reverse Transcriptase (RNaseH-). Second-strand cDNA synthesis was performed using DNA Polymerase I and RNase H. After adenylation of the 3′ ends, the DNA fragments were ligated to NEBNext adaptors with hairpin loop structure for hybridization. The library fragments were purified with AMPure XP system (Beckman Coulter, Beverly, United States) in order to select the short cDNA fragments of 150∼200 bp in length. PCR was performed with Phusion High-Fidelity DNA polymerase, universal PCR primers and index (X) primer. At last, all samples were purified (AMPure XP system) and library quality was assessed using the Agilent Bioanalyzer 2100 system.

### Data Analysis of RNA-Seq and Bioinformatics Analyses

Raw reads of fastq format were firstly processed through in-house perl scripts. For the gene expression level, HTSeq v0.6.1 was used to count the number of reads mapped to each gene. The FPKM (fragments per kilobase of exon per million fragments mapped) of each gene was calculated based on the length of the gene and read count mapped to this gene. FPKM considers the effect of sequencing depth and gene length for the read count at the same time, and it was currently the most commonly used method for estimating gene expression levels (Trapnell et al., 2009). The proportion of mapped reads for each sample were shown in Table 1. DESeq R package (1.18.0) was based on a model using a negative binomial distribution to estimate the variance-mean dependence and differential expression of the count data from high-throughput sequencing analysis (Anders and Huber, 2010). Genes with a log_2_FC value above 1 or below −1 and with an adjusted *P*-value < 0.05 were designated for differential expression. GOseq R package was used in identifying Gene Ontology (GO) terms to annotate enriched genes with corrected *p*-value of less than 0.05. All data were analyzed using the online platform of Novomagic Cloud Platform^1^.

**TABLE 1:** Statistics of reads that mapped to *H. alvei* H4 genome per sample analyzed.

Sample*	W2-1	W2-2	W2-3	W12-1	W12-2	W12-3	IR-1	IR-2	IR-3
Total mapped reads (%)	97.82	97.59	97.89	98.73	98.7	98.88	98.8	98.81	98.82
Uniquely mapped reads (%)	96.93	96.48	97.09	97.81	97.84	98.02	97.95	98.09	98.16
RNA integrity number (RIN)	10	10	10	9.9	8.1	9.8	9.4	8.8	9.8

*Samples W2-1, W2-2, and W2-3 represent biological replicates for RNA samples isolated from *H. alvei* H4 at OD_600_ = 0.3; samples W12-1, W12-2, and W12-3 are biological replicates for RNA isolated from *H. alvei* H4 at OD_600_ = 1.7; samples IR-1, IR-2, and IR-3 represent biological replicates for RNA isolated from Δ*luxIR* at OD_600_ = 1.7. OD_600_, Optical density at 600 nm; IR, both *luxI/R* genes were mutated; W, wild-type.

### qRT-PCR

Quantitative RT-PCR with SYBR Green was used for the analysis of gene expression. Total RNA (30 μl from tested tissues) was converted to cDNA using a PrimeScript^TM^ RT Reagent Kit with gDNA Eraser (TaKaRa, Dalian, China). Real-time quantitative PCR was performed in a 25 ul reaction using a BIO-RAD MyiQ^TM^2 Real Time Detection System and SYBR Green PCR Master Mix (TaKaRa, Dalian, China). The 16S gene was used as an endogenous control. All reactions were carried out in triplicate. The threshold cycle (Ct) was defined as the cycle number at which the fluorescence intensity passed a predetermined threshold. Each gene was quantitated relative to 16S endogenous control using the equation: *N* = 2^–ΔΔCt^ (Livak and Schmittgen, 2001).

### Swimming Assays

We determined swimming according to the method described by Kim et al. (2007) with some modifications. Briefly, each strain was incubated in LB medium until the OD_600_ nm of the cultures reached the indicated value. An aliquot (4 μl) of each cell suspension was spotted onto the center of the motility agar (3 g/l agar, 10 g/l tryptone, 2.5 g/l NaCl). The ability of motility was assessed by measuring the diameter of the zone spread from the point of inoculation at indicated time, and by calculating diffusion rate using the equation: $\mathrm{Diffusion}\,\mathrm{rate}=\frac{D_{t+12}-D_{t}}{D_{t}}$, where *D*_*t*_ was the diameter of target strains at the time *t*.

### Accession Number(s)

The raw and processed transcriptome data of *H. alvei* H4 have been deposited at the Gene Expression Omnibus (GEO) database under the accession number GSE137815.

## Results and Discussion

### Identification of Sampling Points

**FIGURE 1:**
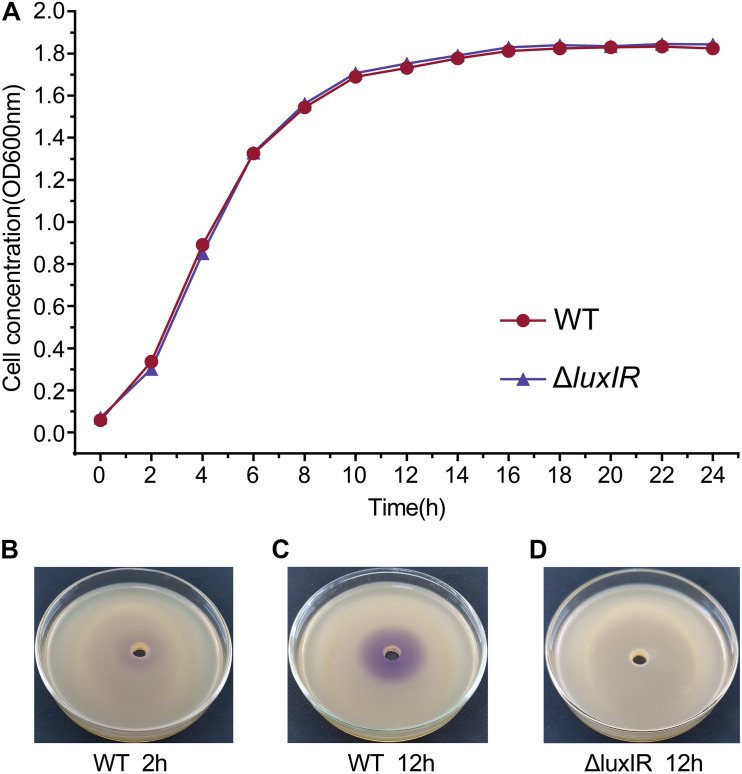
Growth curves and detection of AHLs of *H. alvei* H4. Growth curves of *H. alvei* H4 wild-type (WT) and Δ*luxIR* strain **(A)**. The CV026 agar plates inoculated with 2-h culture supernatant of WT **(B)**, 12-h culture supernatant of WT **(C)**, and 12-h culture supernatant of Δ*luxIR* **(D)**. Filled red circles indicate WT; filled purple triangles indicate Δ*luxIR* strain.

We determined sampling points based on the growth curves and the detection of AHLs by pigment production, which is regulated by QS. The growth curves showed that the cultures of *H. alvei* H4 WT were in the early logarithmic phase (OD_600_ of 0.3) and the early stationary phase (OD_600_ of 1.7) at 2 and 12 h, respectively, so we established these time points as the GPDM RNA sampling points (Figure 1A). In addition, the deletion of *luxI/R* genes have no effect on the growth of *H. alvei* H4, meaning that biomass did not contribute to transcriptome differences between GPDM samples. Pigment production assay indicated that the cultures of *H. alvei* H4 WT produced detectable amounts of signal molecules at 2 h (Figure 1B), while the biosensor plates produced more violet zone when incubated with the ethyl acetate extract prepared from the culture supernatant of WT strain but not from the culture supernatant of Δ*luxIR* at 12 h (Figures 1C,D), indicating that more signal molecules were accumulated in the WT cultures and Δ*luxIR* did not produce signal molecules. Hence, we choice this time point as the *luxI/R*-mediated QS RNA sampling points.

### Differentially Expressed Genes (DEGs) in Responses to GPDM and *luxI/R*-Mediated QS

**FIGURE 2:**
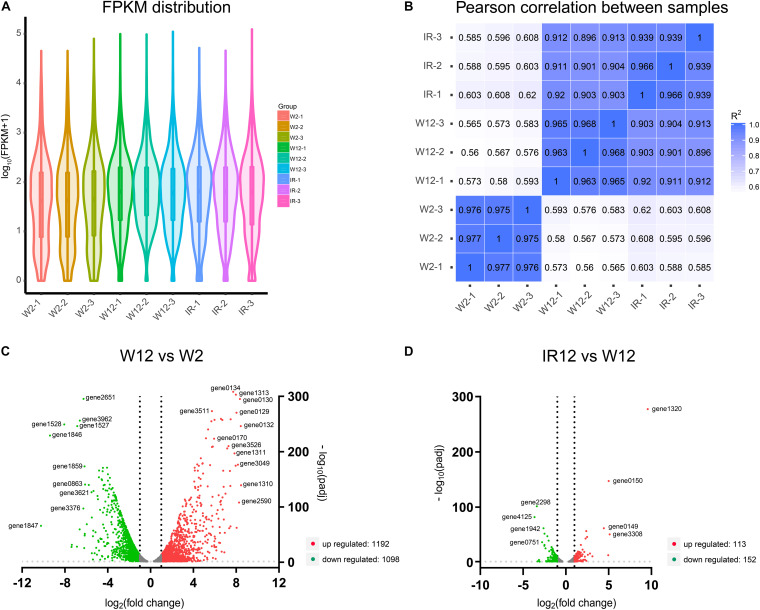
Analysis of gene expression levels in the transcriptome of different samples. **(A)** The violin map of all transcript FPKM values for the nine samples. **(B)** Correlation among nine samples. **(C,D)** Volcano plots of differentially expressed genes of W12-W2 group and IR12-W12 group. The *x*-axis and *y*-axis represent the log_2_(fold change) and -log_10_padj, respectively. Upregulated genes were shown in red and downregulated genes were in green.

In order to elucidate the gene expression patterns and affected metabolic pathways, and to gain further insight into the comprehensive regulatory roles of the full set of genes of *H. alvei* H4 between GPDM and *luxI/R*-mediated QS, transcriptome profiling through RNA-sequencing (RNA-seq) was used and two cases of analyses were specifically adopted. The violin map of FPKM (Figure 2A) for the nine samples in this experiment indicated that all transcript samples had high gene expression levels. Besides, each replicate of the independent treatment showed an extremely high correlation with its respective counterpart (Figure 2B), which indicated the high reproducibility of transcript samples. The comparison (W12/W2) list of DEGs were given in Supplementary Table 1, which includes 2290 differentially expressed genes. Among them, 1192 genes were downregulated, and 1098 genes were upregulated. In the second case of *luxI/R*-mediated QS, 265 genes were differentially expressed (113 genes were upregulated, and 152 genes were downregulated) in strain Δ*luxIR* relative to WT (Supplementary Table 2). According to the volcano plots, the DEGs were either downregulated (green dots) or upregulated (red dots) by more than twofold (Figures 2C,D). We could preliminarily conclude that the regulation of GPDM was more complex than that of *luxI/R*-mediated QS at the transcriptional level, indicating GPDM involved in more metabolic pathways.

### Real-Time Quantitative PCR Validation

**FIGURE 3:**
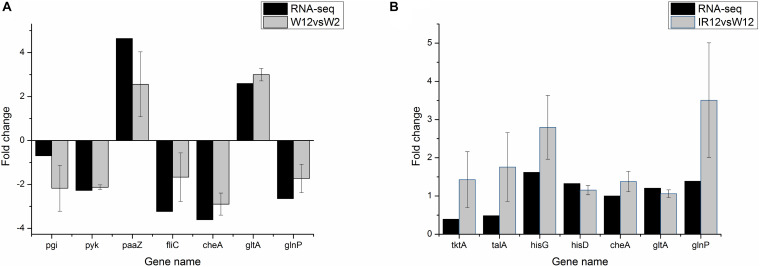
qRT-PCR validation of differential gene expression profiles obtained from RNA-seq. qRT-PCR was performed in triplicate for genes selected from the pathways controlled by GPDM **(A)** and luxI/R-mediated QS **(B)**. In the qRT-PCR analysis, the relative fold difference in gene expression was calculated by the 2^– Δ^ ^Δ^ ^CT^ method using the 16S rRNA gene as an internal reference. For comparison with RNA-seq data, the log_2_2^– Δ^ ^Δ^ ^CT^ values were calculated and the data was analyzed using Origin version 8.5.1. The positive and negative values of the log_2_(fold change) indicate upregulation and downregulation, respectively.

To verify the differential gene expression obtained by RNA-seq, a total of 11 genes were selected from DEGs (including *fliC*, *paaZ*, *pgi*, *pyk* from the W12-W2 group; *tktA*, *talA*, *hisG*, *hisD* from the IR12-W12 group and *cheA*, *gltA*, *glnP* from the section co-regulated by the both group) for quantitative reverse transcription polymerase chain reaction (qRT-PCR) analysis. As shown in Figure 3, *pgi*, *pyk*, *cheA*, and *glnP* in the W12-W2 group were downregulated by about 2-fold whereas *paaZ*, *fliC*, and *gltA* were upregulated, while all seven genes in the IR12-W12 group were upregulated. The RNA-seq data obtained from the expression patterns of these 11 genes were largely confirmed by the qRT-PCR data, which verified the accuracy of the mRNA differential expression data in *H. alvei* H4.

### GO and KEGG Pathways Enrichment Analysis of DEGs in Response to GPDM and *luxI/R*-Mediated QS

In the first situation, the genomic transcription levels between W12 and W2 were compared to further understand which metabolic pathways regulated by GPDM. DEGs of the GO and KEGG pathway categories were examined. The GO category was found to include Biological Processes (BP) that involved in cellular process, metabolic process, biological process and cellular component organization or biogenesis. The GO category also included Molecular Function (MF) that mainly consists of catalytic activity, single-molecule function, transporter activity and binding. The percentage of Cellular Component (CC) classification, another GO category, was relatively weak compared with W2 (Supplementary Figure 1). DEGs were also clustered in the KEGG pathways to elucidate the metabolic pathways that involve DEGs in their regulation. The overexpressed KEGG pathways were those associated with ABC transporters, microbial metabolism in di-environments, butanoate metabolism, pyrimidine metabolism, phenylalanine metabolism, and glycolysis/gluconeogenesis (Supplementary Figures 2, 3).

In the second situation, the levels of IR12 and W12 transcription were compared to further understand the function of the QS system on the regulation of metabolic pathways. The classification of GO and KEGG pathways was also performed for this group of DEGs. Compared with W12, metabolic process, localization, cellular process and response to stimulus were the most enriched groups in the category of Biological Process (BP) in IR12, while the specific classifications in the Cellular Component (CC) and Molecular Function (MF) categories were almost identical to the GPDM (Supplementary Figure 4). The KEGG enrichment analysis revealed significantly enriched DEGs in most KEGG pathways. Among them, the most significantly downregulated metabolic pathways were involved in starch and sucrose metabolism, butanoate metabolism and two-component system, whereas the significantly upregulated metabolic pathways include those involved in histidine metabolism, carbon metabolism, bacterial chemotaxis, and citrate cycle (TCA cycle) (Supplementary Figures 5, 6). The plot shows the most significantly enriched 20 pathway entries (Supplementary Figures 2, 3, 5, 6). For those with less than 20 enriched path entries, all were displayed.

### Significantly Enriched Pathways in *H. alvei* H4 Co-regulated by *luxI/R*-Mediated QS and GPDM

**FIGURE 4:**
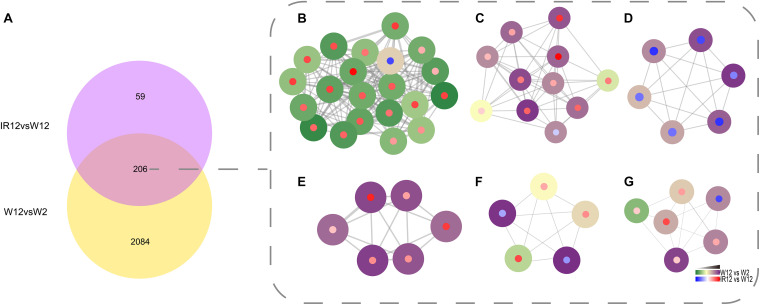
Identification of the QS regulon co-regulated by GPDM and *luxI/R*-mediated QS. Venn diagram of differentially expressed genes in the W12-W2 group and IR12-W12 group **(A)**. Six top gene clusters identified by the MCODE in Cytoscape, which were co-regulated by GPDM and *luxI/R*-mediated QS, involved in chemotaxis **(B)**, citrate cycle **(C)**, two-component system **(D)**, ABC transporters **(E)**, pyruvate **(F)**, and butanoate **(G)** metabolism, respectively. Each node represents a unique gene. Genes regulated by GPDM (Periphery of the node), by *luxI/R*-mediated QS (Center of the node) were shown. The thickness of a line connecting two nodes was an indicator of functional similarity.

To identify genes that were co-regulated by the above density-dependent regulation, we analyzed the RNA-seq data of *luxI/R*-mediated QS compared to the corresponding GPDM. The results were visualized in Cytoscape 3.8.0 with the MCODE package (Figure 4), which showed the number of DEGs that overlap between the *luxI/R*-mediated QS and GPDM transcriptome and function in chemotaxis, citrate cycle (TCA cycle) and other significantly enriched pathways as follows.

#### Chemotaxis

**FIGURE 5:**
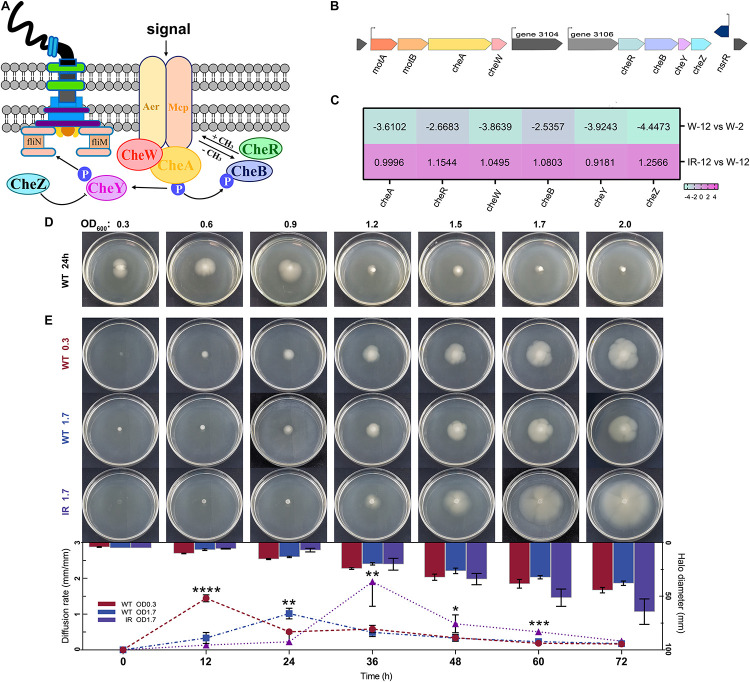
Chemotaxis clusters organization and swimming phenotype of *H. alvei* H4. **(A)** Schematic representation of the central chemotaxis apparatus. **(B)** Genomic organization of major gene clusters for chemotaxis in *H. alvei* H4. **(C)** Heat map of genes involved in the chemotaxis clusters. **(D)** The swimming phenotype of *H. alvei* H4 wild-type strain (WT) at indicated OD_600_ value on motility agar plates at 30°C for 24 h. **(E)** The swimming phenotype of OD_600_ 0.3 WT, OD_600_ 1.7 WT and OD_600_ 1.7 Δ*luxIR* (marked as WT 0.3, WT 1.7 and IR 1.7, respectively) photographed every 12 h intervals until 72 h after inoculation on motility agar plates (Plates section). Diffusion rate of *H. alvei* H4 WT and Δ*luxIR* (Histogram and line graph section). The diameter of opalescent zones was included in the histogram, as obtained using the proposed method. Line graphs shown the swimming rate of the target strains. Filled red circles indicate WT 0.3; filled blue squares indicate WT 1.7; filled purple triangles indicate IR 1.7. Data were the means ± SEMs (*n* = 3). Different asterisks above the node indicate significant differences at the *P* < 0.05 level.

Chemotaxis is the movement of an organism in response to a chemical stimulus (Micali and Endres, 2016). The chemotactic signals are sensed by the methylation-expressing protein (MCP) and transduced to the sensory histidine kinase CheA through the scaffolding protein CheW. CheA∼P serves as a phophodonor to the CheY and CheB response regulators, which activate and inhibit chemotactic response, respectively (Figure 5A). The genomic organization of major gene clusters for chemotaxis in *H. alvei* H4 was presented in Figure 5B. In the IR12-W12 group, almost all upregulation involved in bacterial chemotactic genes indicated the limitation of *H. alvei* H4 chemotaxis imposed by the *luxI/R*-mediated QS at the mRNA level (Figure 5C). In addition, the expression of a gene encoding MCP, a transmembrane protein involved in the detection and transduction of extracellular sensory signals (Derr et al., 2006), was also partially upregulated. It was not surprising that all genes involved in the bacterial chemotaxis in the W12-W2 group were downregulated by more than 2.5-fold, most probably due to weak chemotactic sensing (Colin et al., 2019), consequently limiting the expression of chemotactic genes. In addition, the chemotactic gene, *cheA*/*cheY*, which were involved in extracellular signal transduction (Dons et al., 2004), were upregulated in the IR12-W12 group but were downregulated by more than 3-fold in the W12-W2 group, consistent with the trend displayed by members of the gene family involved in chemotaxis and the two-component system.

To determine whether these regulatory mechanisms cause the same trend in the swimming phenotype, the development of opalescent zones around the target colonies was measured in two treatments: (1) The wild-type strains at indicated cell densities were applied to motility agar plates at 30°C for 24 h; (2) The wild-type strains and Δ*luxIR* harvested at different OD values (0.3 or 1.7) were spotted on motility agar plates for 72 h. We observed that the diameter of the zones gradually decreases as the OD value increases when OD_600_ exceeds 0.9 in the first treatment group (Figure 5D), and that swimming phenotype of wild-type strain at initial OD_600_ of 0.3 (WT 0.3) was the first to be activated at 12 h and the diffusion rate at this time was 4 to 5 times greater than that of wild-type strain at initial OD_600_ of 1.7 (WT 1.7) in the second treatment group (Figure 5E), indicating that cell density seems to affect negatively the swimming motility. Compared to wild-type strain, there was a delay in the initiation of motility of Δ*luxIR* at initial OD_600_ of 1.7 (IR 1.7) until 36 h but it was clear that this strain possesses a higher diffusion rate when activated, almost 4-fold higher than that from the WT 1.7 at 36 h, thereby generating a larger diameter relative to the WT 1.7 at 72 h. Interestingly, we found that the swimming phenotype of the cells harvested at different cell densities were diverse, which indirectly reflected the expression difference of some genes detected in the transcriptome. Additionally, the target strains had the same metabolic state at the beginning of the experiment. However, this metabolic synchronization may be broken due to the different factors of the swimming experiment, GPDM and *luxI/R*-mediated QS, which could interfere with some metabolic pathways during the culture process. Eventually, differences in swimming phenotype-related genes and metabolism may lead to different swimming phenotypes. Nonetheless, how the expression of certain genes to affect the swimming behavior of cells during the growth curve maintained in the plates remains unknown. Thus, more work may be needed to explore the swimming behavior-related signaling pathways, especially changes in signal transmission caused by genetic differences.

#### TCA Cycle

As a central metabolic pathway, the TCA cycle is a major source of energy supply and anabolic intermediates. The eight enzymes in the TCA cycle catalyze a series of reactions that oxidize the acetyl group of acetyl-CoA to CO_2_, and in most bacteria, electrons are transferred to hydrazine, NAD^+^ and NADP^+^ (Liu et al., 2018). Furthermore, the TCA cycle also provides precursors such as α-ketoglutarate and oxaloacetate for the biosynthesis of amino acids. Alpha-ketoglutarate could be converted to glutamate and it plays an important role in regulating the balance between carbon and nitrogen metabolism in most microorganisms (Yovkova et al., 2014). Different from the negative regulation observed for the chemotaxis pathway, the GPDM and *luxI/R*-mediated QS were found to exert a positive regulation on the TCA cycle. In the IR12-W12 group, the enzymes of the TCA cycle were upregulated as a result of the deletion of the *luxI/R* gene, and the expression levels of the *icd* and *gltA* genes (both of which were among the differentially expressed genes) were upregulated 2.4-fold and 1.2-fold, respectively. The upregulation of these differentially expressed genes indicated that the *luxI/R*-mediated QS might exert a negative regulatory effect on the TCA cycle at the transcription level. In W12-W2, the TCA cycle was enhanced by the GPDM, indicating a positive adjustment of the TCA cycle. The transcription level of the *gltA* gene was upregulated 2.6-fold in W12, which may result in an increase in intracellular glutamate that can be used for amino acid biosynthesis by transamination. The expression levels of the *sucAB* and *sdhABCD* genes in W12 increased by about 2- to 3-fold, while no significant change in the expression level of the *icd* gene was observed. One possible reason for the opposite trend displayed by the GPDM and *luxI/R*-mediated QS in the regulation of the TCA cycle could be that thousands of differentially expressed genes detected during the exponential growth phase might not be affected only by cell-density dependent behavior, but also by other factors (Patzelt et al., 2013; Puri et al., 2017). As described in a previous study, the expression of many genes may be turned off while other sets of genes may be turned on over the various growth phases (Han et al., 2017).

#### Other Significantly Enriched Pathways

ABC transporters utilized ATP energy to transport inorganic ions, amino acids, hydrocarbons, polypeptides or hydrophobic compounds to achieve transmembrane transport (Nicolaou et al., 2010). In bacteria, ABC transporters catalyzed the uptake of essential nutrients or the extrusion of toxic substances (Davidson and Chen, 2004). It was found that among the common differentially expressed genes of GPDM and *luxI/R*-mediated QS, many of the upregulated genes were those encoding phosphate and amino acid transporters as well as peptide/nickel ABC transporters (corresponding to the “transport activity” in the GO pathway). The expression of GPDM regulatory genes, measured in fold change, was higher than that of *luxI/R*-mediated QS (data not shown). GPDM and *luxI/R*-mediated QS genes were also found to be involved in the regulation of butanoate and pyruvate metabolism, as well as the two-component systems. Two-component system (TCS) was an important molecular device for sensing and transducing environmental signals (Stock et al., 2000; Roux et al., 2009). It enables bacteria to sense, respond, and adapt to a wide variety of environmental signals and growth conditions, and to respond to a wide range of stimuli such as antibiotics, nutrients, cellular redox state, quorum signals, chemoattractants, temperature and pH (Attwood et al., 2007).

In addition to their co-regulated functions described above, it was clear that they shown differences in the regulation of other metabolic pathways, indicating the complementarity of metabolic regulation and the complexity of QS regulation process affected by many other factors, not just cell density (Zhao et al., 2014; Feng et al., 2015; Goo et al., 2015).

### Significantly Enriched Pathways Regulated by GPDM in *H. alvei* H4

#### Phenylalanine Metabolism

**FIGURE 6:**
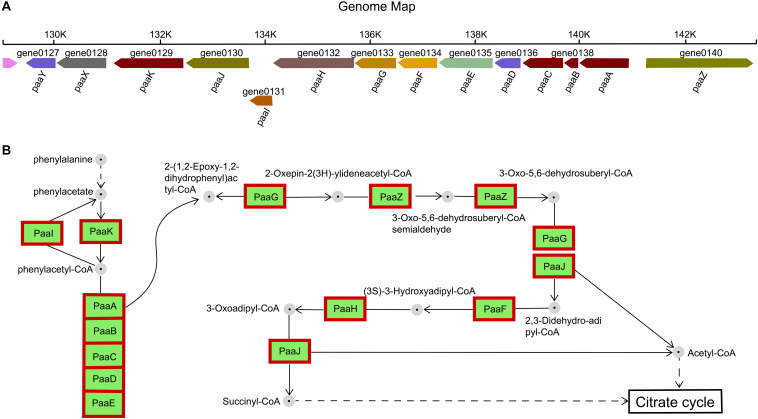
Phenylacetate catabolic pathway. **(A)** Catabolic gene cluster for phenylacetate degradation in *H. alvei* H4. **(B)** Reactions and intermediates of the pathway in *H. alvei* H4. In the genomic map, the number below the gene arrow was the locus tag corresponding to the genome and different colors represent the predictive functions of different genes. Proteins appear within red frames were upregulated at the mRNA level.

The gene cluster encoding the phenylalanine catabolic pathway of *H. alvei* H4 consist of 14 genes organized in ten contiguous operons: *paaY*, *paaX*, *paaK*, *paaJ*, *paaI*, *paaHGF*, *paaE*, *paaD*, *paaCBA, and paaZ* (Figure 6A). In *H. alvei* H4, these 14 genes were found to correspond to five specific COG-type descriptions: (i) Transcription (paaX); (ii) Secondary metabolites synthesis (paaA, paaB, paaC, aaK, and paaI); (iii) Energy production and conversion (paaE); Lipid transport and metabolism (paaJ, paaH, paaG, paaF, and paaZ); (v) Unknown function (include Transferase paaY and fes assembly suf system protein paaD).

The functions of the 14 enzymes involved in phenylalanine metabolism were mainly related to assembly, catalysis, and regulation. Previous studies have designated PaaABC(D)E as the first member of the family of bacterial di-iron multicomponent oxygenases, which acts on CoA-esters, and PaaD has been suggested to be part of the oxygenase complex, possibly acting as a stabilizing element (Notomista et al., 2003; Fernandez et al., 2006). The paaD-related gene was adjacent to the cysteine desulfurase gene *sufS*, which corresponds to the description of the COG-type of PaaD in the *H. alvei* H4 genome, and its likely role could be in the assembly of iron sulfur clusters, although the actual function was unknown. The β-oxidation steps leading to acetyl-CoA and succinyl-CoA (metabolites of the tricarboxylic acid (TCA) cycle) were catalyzed by PaaJ, PaaF, and PaaH (Figure 6B), and the product of sulfolytic cleavage of 3-oxo-5,6-dehydrosuberyl-CoA by PaaJ was 2,3-Didehydroadipyl-CoA, rather than the expected 3,4-dehydroadipyl-CoA. This 3,4-2,3 isomerization reaction may be catalyzed by 2-(1,2-epoxy-1,2-dihydrophenyl) acetyl-CoA isomerase PaaG (Teufel et al., 2010). Furthermore, PaaX was classified as transcription in the COG-type description, and PaaX acts as a transcriptional repressor in *E. coli*, while phenylacetyl-CoA acts as a specific inducer that prevents PaaX from binding to its target sequence (Ferrandez et al., 2000). PaaY (a protein of unknown function whose specific COG type was described as Transferase) was located in the same transcription unit as PaaX, so it seems likely to play a regulatory, possibly inactivating PaaK (a phenylacetate-CoA Ligase) through acetylation. Therefore, PaaY could downregulate the degradation of phenyl acetate at the enzyme level, rapidly performing metabolic regulation in response to increased levels of acetyl-CoA (Teufel et al., 2010). In the RNA-seq experiment, all 14 genes showed an upregulation of greater than 6-fold, indicating that the condition of HCD significantly enhanced the metabolism of phenylalanine.

#### Glycolysis/Gluconeogenesis

**FIGURE 7:**
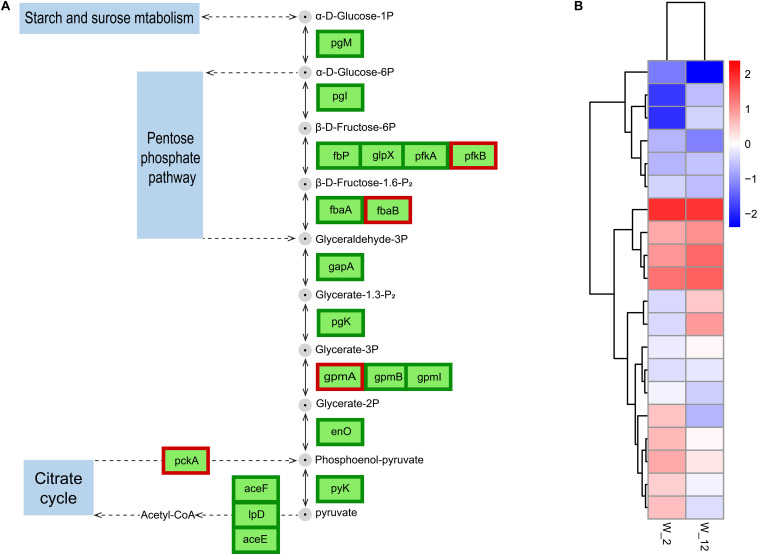
Expression of GPDM altering genes involved in glycolysis/gluconeogenesis. **(A)** Differentially expressed genes (DEGs) involved in glycolysis/gluconeogenesis. **(B)** Normalization of gene expression to the heat map with W2 as a control. Proteins enclosed in green boxes and red boxes were downregulated and upregulated, respectively, at the mRNA level.

Glycolysis produces energy and is therefore essential for cell growth. This process has been studied more frequently in plant germination and seedling growth (Wang et al., 2015). The regulation of wild-type *H. alvei* H4 growth by GPDM was found to be closely related to glycolysis through the overall analysis of transcripts in W12 and W2, where glycolysis in W12 was found to be less active than in W2 (Figure 7), most probably due to the lack of nutrients caused by HCD. As reported by Joyner et al. (2016), many organisms adopt a dormant state at HCD, in which their metabolism slows down to conserve vital energy to survive in harsh conditions, while simultaneously permissive to essential biochemistry. In *H. alvei* H4, a weakening of this process in the case of W12 caused a decrease in electron flux which may not produce sufficient NAD^+^, leading to decreased cellular metabolism. In addition, the genes encoding the glycolytic rate-limiting enzymes, including glucose-6-phosphate isomerase (*pgi*) and pyruvate kinase (*pyk*), were further confirmed by qRT-PCR analysis (Figure 3). Phosphofructokinase and pyruvate kinase were important control points in the glycolytic pathway because they catalyzed two irreversible steps. Pyruvate kinase catalyzed the final step of glycolysis in which pyruvate and ATP were formed (He et al., 2019). According to the RNA-seq data, GPDM might exert significant regulation on the glycolysis/gluconeogenesis pathway, which corresponded to a decrease in energy required for the growth of WT from 2 h to the 12 h.

### Significantly Enriched Pathways in *H. alvei* H4 Regulated by *luxI/R*-Mediated QS

#### Pentose Phosphate Pathway

**FIGURE 8:**
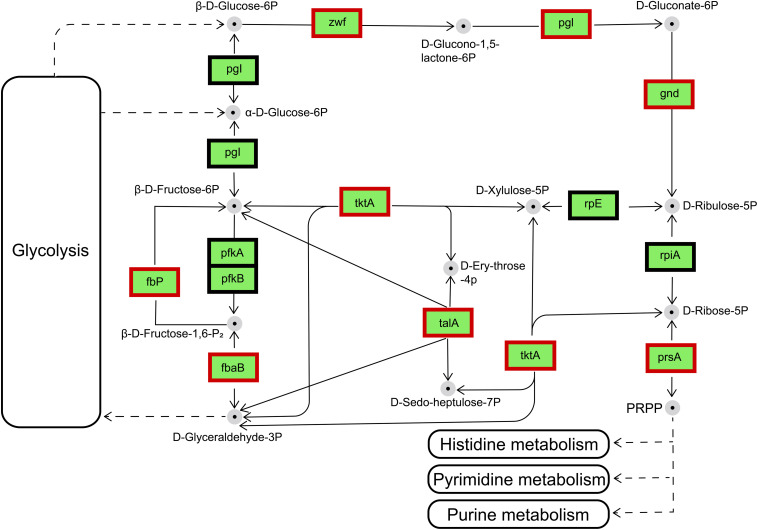
Regulation of the genes involved in the pentose phosphate pathway by the differentially expressed genes in IR12-W12. Genes appearing within red frames were upregulated, and genes appearing within black frames showed no significant changes.

The main biochemical function of the pentose phosphate pathway (PPP) is the synthesis of nucleic-acid and amino-acid sugar-phosphate precursors, which are essential to the anabolic processes in the cell and to the control and maintenance of its redox homeostasis (Stincone et al., 2015). The transcriptional changes occur in a fully coordinated manner, and enzymes were subsequently induced according to their molecular function (Ihmels et al., 2004; Chechik et al., 2008). Details of the transcriptional regulation of the PPP enzymes vary widely among different organisms. Therefore, only the main mechanisms will be discussed here. In the IR12-W12 group, transcriptome data showed that the phosphofructokinase gene was not expressed and the Embden-Meyerhof-Parnas pathway (EMP) was disturbed in the Δ*luxIR* mutant (data not shown). Thus, glucose and gluconate may be phosphorylated by glucose and gluconate kinases, respectively, and further catabolized via PPP (Deppenmeier et al., 2002; Kersters et al., 2006; Deppenmeier and Ehrenreich, 2009). The differentially expressed genes encoding enzymes involved in pentose phosphate metabolism were upregulated in Δ*luxIR.* These enzymes were glucose-6-phosphate 1-dehydrogenase [*zwf*, gene1257, (EC: 1.1.1.49/1.1.1.363)], 6-phosphogluconolactonase [*pgl*, gene1256, (EC: 3.1.1.31)], 6-phosphogluconate dehydrogenase [*gnd*, gene3434, (EC: 1.1.1.44/1.1.1.343)], ribose-phosphate pyrophosphokinase [*prsA*, gene3207, (EC: 2.7.6.1)] Transketolase (TKL) [*tktA*, gene2203, (EC: 2.2.1.1)], transaldolase (TAL) [*talA*, gene1309, (EC: 2.2.1.2)], fructose-bisphosphate aldolase, class II [*fbaB*, gene1771, (EC:4.1.2.13)] and fructose-1,6-bisphosphatase II [*fbp*, gene2020, (EC: 3.1.3.11)] (Figure 8). Among these, TKL used a ketose donor (D-Xylulose-5P) and aldose acceptors (D-Ribose-5P or D-Erythrose-4P) to form aldose and ketose products (D-Glyceraldehyde-3P and D-Sedoheptulose-7P or β-D-Fructose-6P, respectively). The donor substrates of TAL are ketose sugar phosphates which include β-D-Fructose-6P and D-Sedoheptulose-7P whereas the acceptor substrates of TAL were the aldose sugar phosphates D-Glyceraldehyde-3P and D-Erythrose-4P (Samland and Sprenger, 2009). By sharing these intermediate metabolites with glycolysis (β-D-Fructose-6P and D-Glyceraldehyde-3P), TAL and TKL act as a bridge between glycolysis and PPP (Chechik et al., 2008).

#### Histidine Metabolism

**FIGURE 9:**
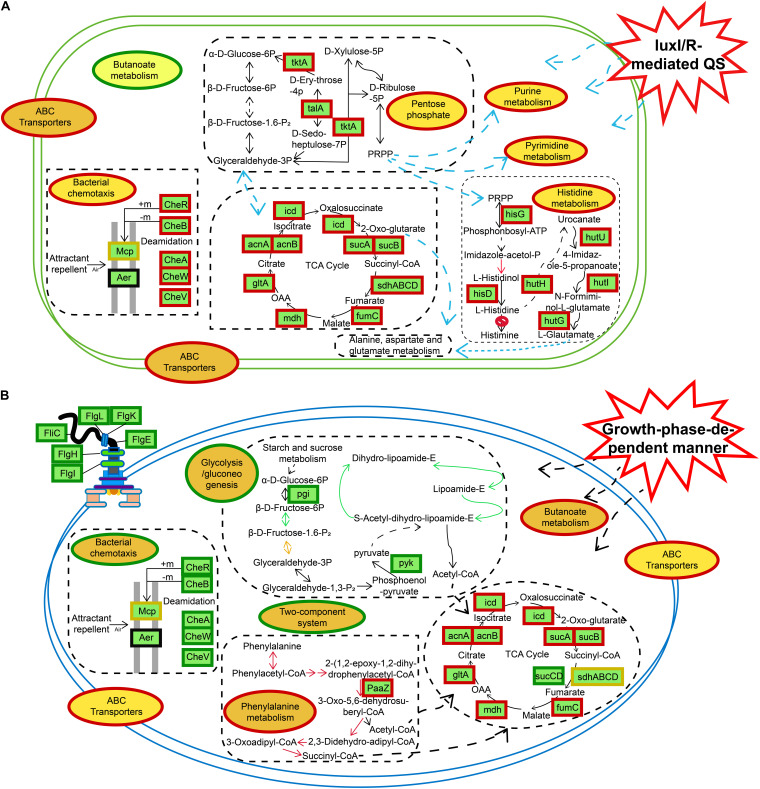
Schematic representation of certain biological pathways in *H. alvei* H4 affected by different situations. **(A)** *luxI/R*-mediated QS. **(B)** GPDM, most DEGs were integrated and were indicated in red borders (upregulated), green borders (downregulated), and yellow borders (upregulated and downregulated), respectively.

In the IR12-W12 group, the loss of *luxI/R* in *H. alvei* H4 increased the expression of 9 of the 12 genes involved in the metabolic pathway of histidine, thereby accelerating the conversion of PRPP from the pentose phosphate pathway to L-histidine. In addition, the RNA-seq data indicated that gene 0518–gene 0525 could be an operon, and some genes were highly and differentially expressed in the IR12-W12 group, including *hisG* (EC: 2.4.2.17), *hisA* (EC:5.3.1.16), and *hisB* (EC:4.2.1.19, EC:3.1.3.15), *hisC* (EC:2.6.1.9), and *hisD* (EC:1.1.1.23) (Figure 9A). The RNA-seq data also revealed a lack of enzymes responsible for the conversion of histidine to histamine (EC: 4.1.1.22) in *H. alvei* H4. This might be because its reference genome was a draft genome, so we could not detect histamine production at the metabolite level. In the IR12-W12 group, the deletion of the *luxI/R* gene also increased the expression of 10 of the 16 genes belonging to the biosynthetic pathways of valine, leucine and isoleucine. Such an impact may help to balance the carbon and electron flow to increase the production of amino acids (Miller et al., 2009; Linville et al., 2013). It could also overcome the weak acid stress to accelerate the metabolism of histidine.

## Conclusion

In the IR12-W12 group, the knockout of the *luxI/R* gene led to the upregulation of the expression of related genes in the pentose phosphate pathway and the TCA cycle, resulting in high metabolism and providing NADPH and a large number of metabolites for a diverse range of synthetic reactions (Wu et al., 2017). Moreover, genes associated with nucleic acid metabolism (purine metabolism, pyrimidine metabolism), histidine metabolism, and bacterial chemotaxis were also upregulated in the mutant Δ*luxIR* (Figure 9A). In addition, GPDM had a negative impact on glycolysis/gluconeogenesis, bacterial chemotaxis, flagellar assembly and the two-component systems (Figure 9B). Bacterial chemotaxis, flagellar assembly and the two-component systems were interconnected and act synergistically, depending on cell density to move the bacteria away from an unfavorable environment, thereby transferring them to the source of the nutrient and triggering a chemotactic response (Porter et al., 2011). According to a study by Joyner et al. (2016), when cell density reaches a certain threshold, the bacteria will grow slowly, and their energy will be spent on essential biochemistry, then from this conclusion we can also infer the downregulation of bacterial chemotactic genes in the HCD. The use of transcriptomics shed light on the specific mechanism of metabolic regulation, and the results from the transcriptomic studies could be used as a blueprint to reveal new potential processes that can then be studied in more detail, observing corresponding phenotypic differences.

## Data Availability Statement

The datasets presented in this study can be found in online repositories. The names of the repository/repositories and accession number(s) can be found in the article/Supplementary Material.

## Author Contributions

HHo, CY, XL, and GZ contributed to the study conception and design. CY, XL, and YZ preformed the material preparation, data collection, and analysis. XL and CY wrote the first draft of the manuscript. All authors commented on previous versions of the manuscript and approved the final manuscript.

## Conflict of Interest

The authors declare that the research was conducted in the absence of any commercial or financial relationships that could be construed as a potential conflict of interest.

## Footnotes

1 https://magic.novogene.com/public/customer/login
