# Supplementary material for: Quorum Sensing-Mediated and Growth Phase-Dependent Regulation of Metabolic Pathways in Hafnia alvei H4
Source: Front Microbiol. 2021 Mar 2;12:567942. doi: 10.3389/fmicb.2021.567942 (PMC7960787; doi:10.3389/fmicb.2021.567942)
Supplement: Supplementary file 1 [file Data_Sheet_1.PDF]

Supplementary Material

Quorum sensing-mediated and growth phase-dependent regulation of metabolic pathways in *Hafnia alvei* H4

Congyang Yan[1,2], Xue Li[1,2], Gongliang Zhang[1,2], Yaolei Zhu[1,2], Jingran Bi[1,2], Hongshun Hao[2], Hongman Hou[1,2*]

*** Correspondence:** Hongman Hou: houhongman@dlpu.edu.cn

Supplementary figures

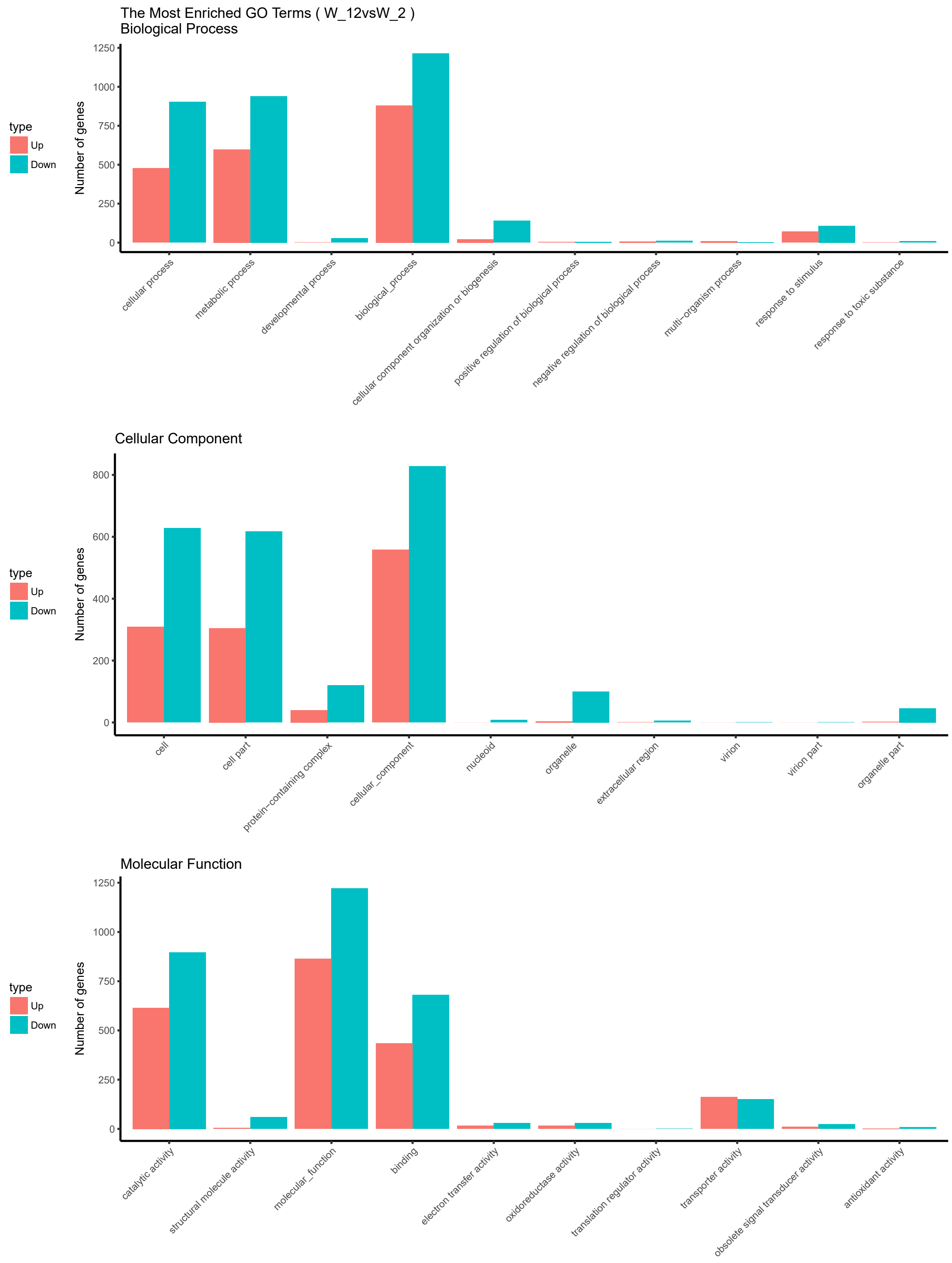

Supplementary Figure 1 | Significantly enriched Gene Ontology categories of differentially expressed genes in W12-W2 group based on RNA-seq analysis.

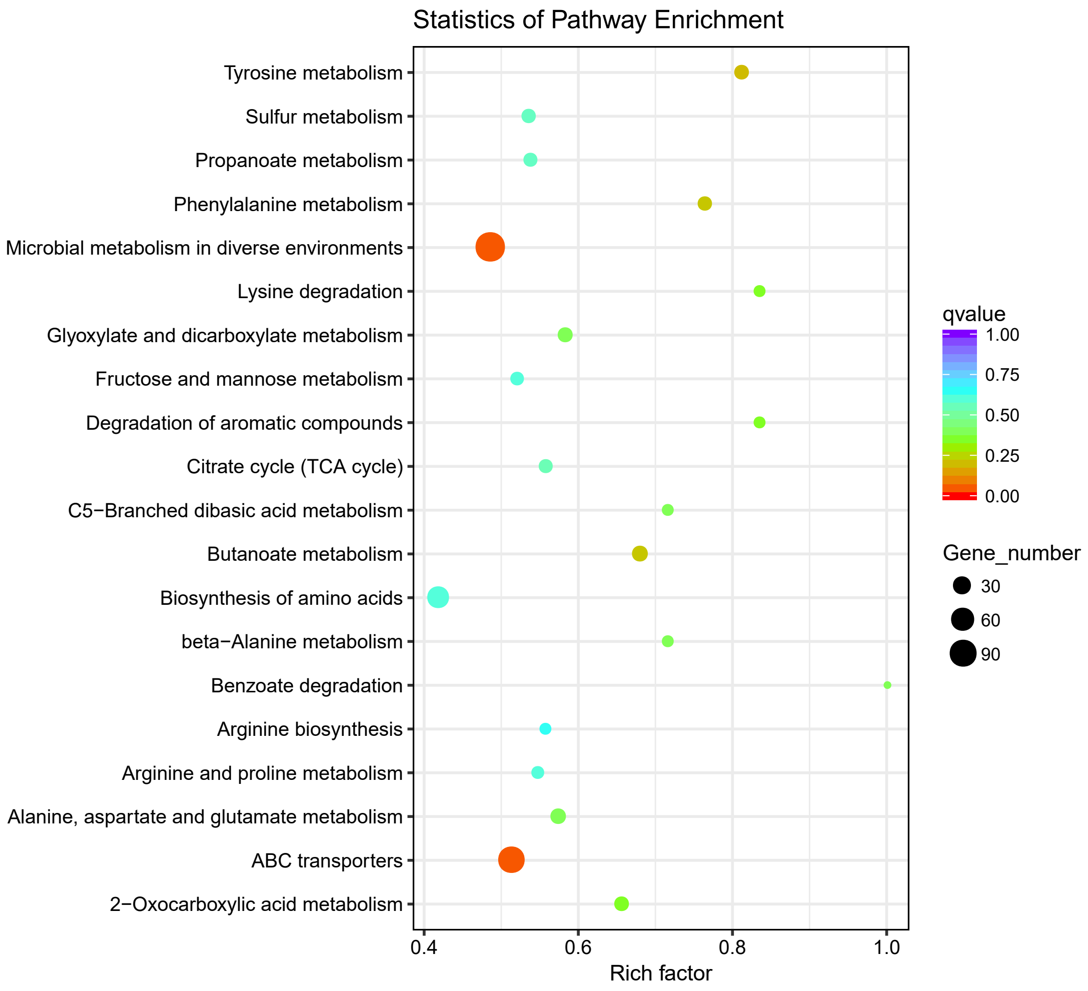

Supplementary Figure 2 | Up-regulated genes in the KEGG pathway scatter plot of differentially expressed genes in W12-W2 group based on RNA-seq analysis.

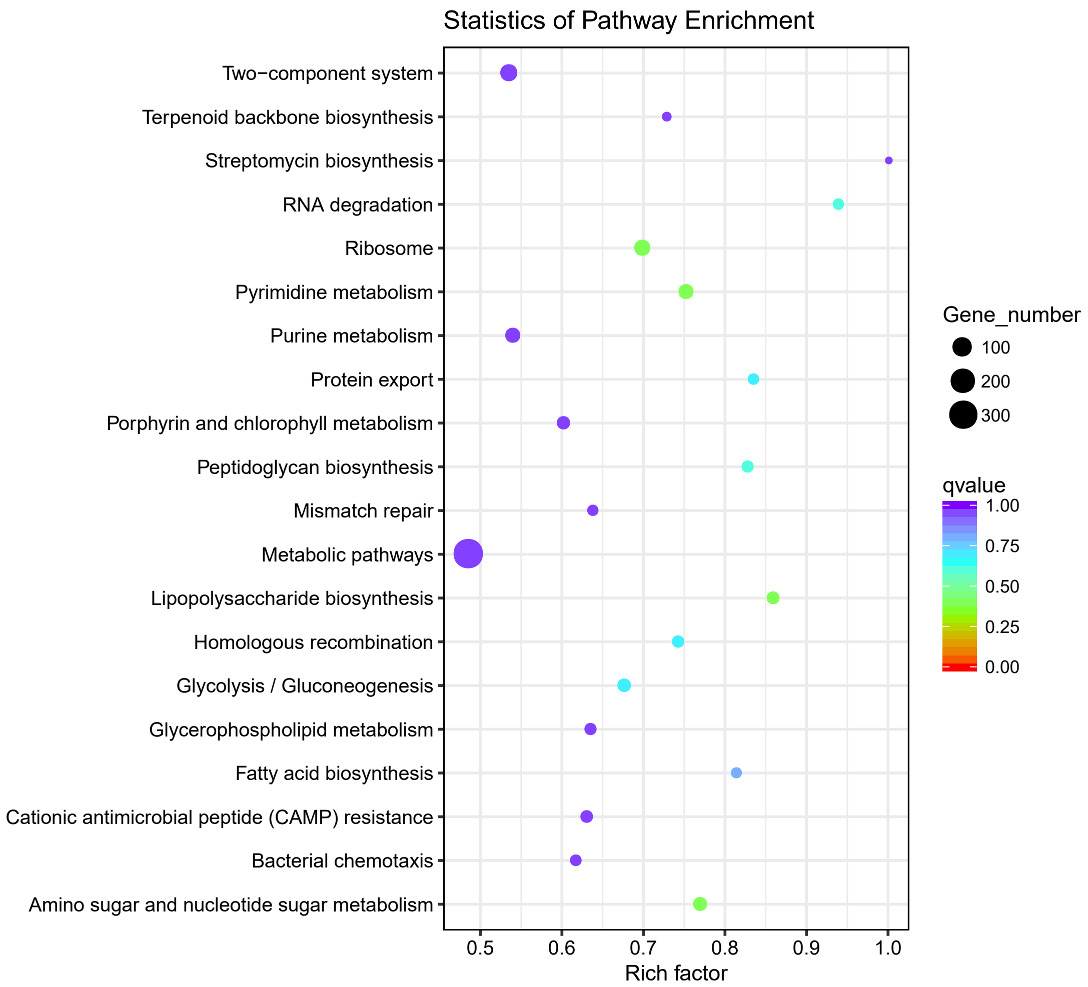

Supplementary Figure 3 | Down-regulated genes in the KEGG pathway scatter plot of differentially expressed genes in W12-W2 group based on RNA-seq analysis.

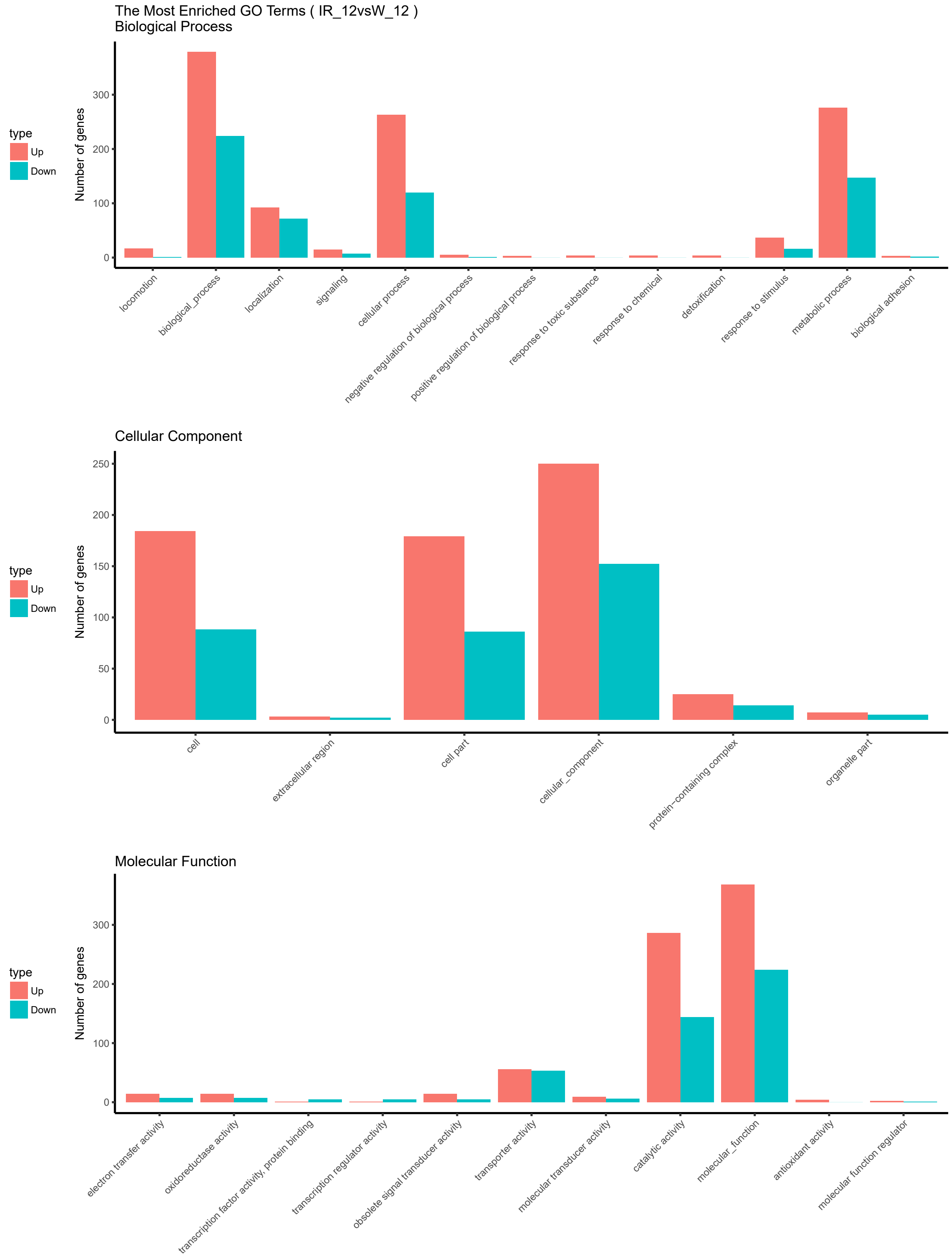

Supplementary Figure 4 | Significantly enriched Gene Ontology categories of differentially expressed genes in IR12-W12 group based on RNA-seq analysis.

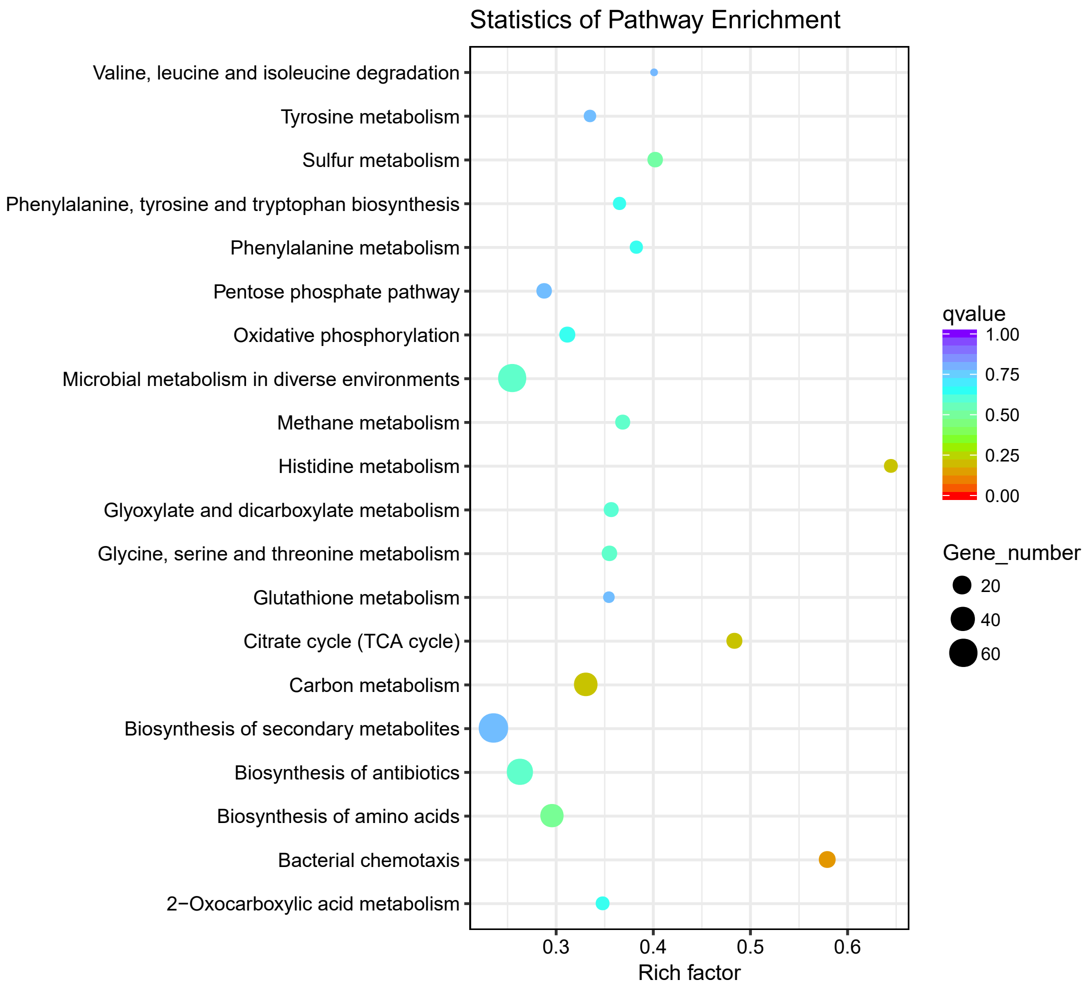

Supplementary Figure 5 | Up-regulated genes in the KEGG pathway scatter plot of differentially expressed genes in IR12-W12 group based on RNA-seq analysis.

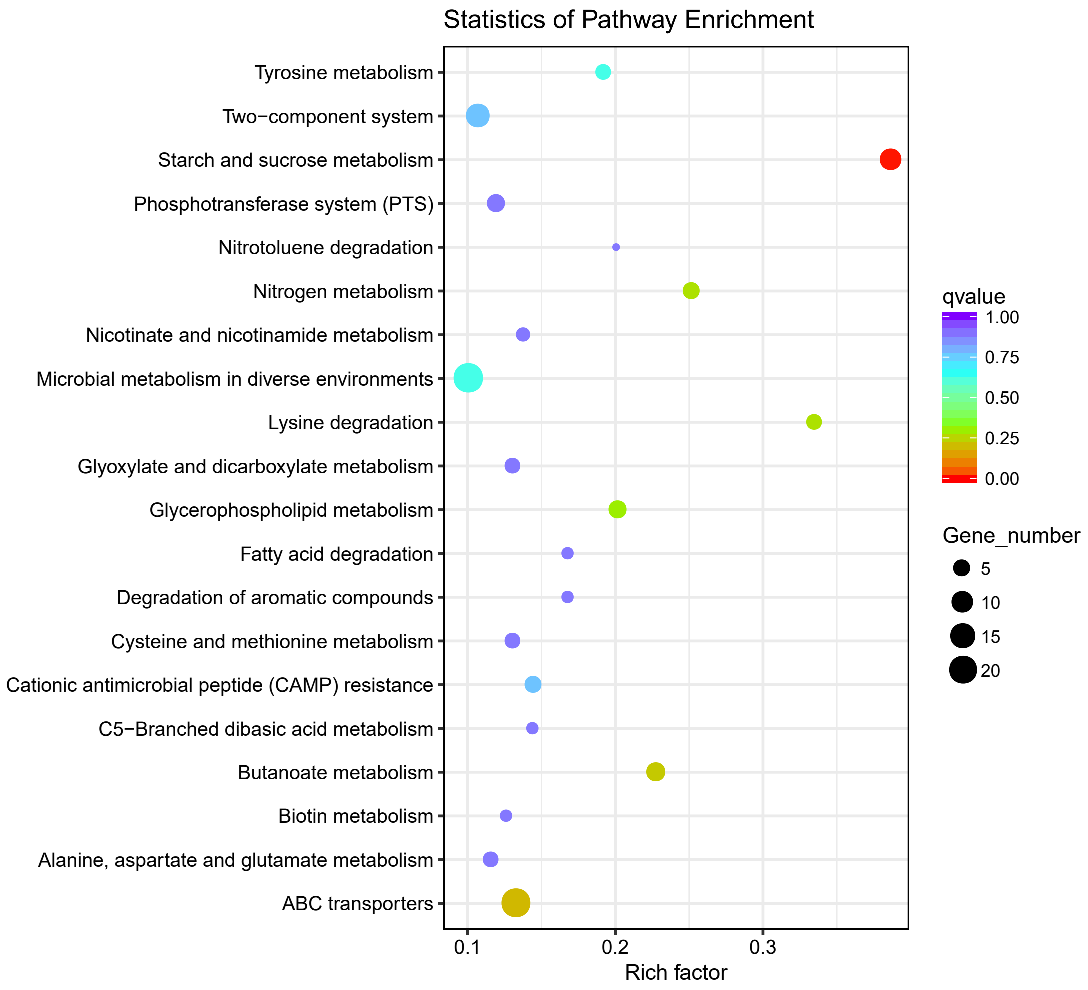

Supplementary Figure 6 | Down-regulated genes in the KEGG pathway scatter plot of differentially expressed genes in IR12-W12 group based on RNA-seq analysis.
